# Differential Expression of Genes Associated with the Progression of Renal Disease in the Kidneys of Liver-Specific Glucokinase Gene Knockout Mice

**DOI:** 10.3390/ijms14036467

**Published:** 2013-03-21

**Authors:** Wei Xu, Hui Li, Rong Wang, Zhen Lei, Yiqing Mao, Xi Wang, Yizhuang Zhang, Tingting Guo, Rongjing Song, Xiaojing Zhang, Ling Jin, Zhixin Li, David M. Irwin, Gang Niu, Huanran Tan

**Affiliations:** 1 Department of Pharmacology, Peking University, Health Science Center, Beijing 100191, China; E-Mails: xuwei@bjmu.edu.cn (W.X.); lihui@bjmu.edu.cn (H.L.); wangrong@bjmu.edu.cn (R.W.); maoyiqing@bjmu.edu.cn (Y.M.); Xixi1125@tom.com (X.W.); onepiecezyz@yahoo.com.cn (Y.Z.); ttguo@bjmu.edu.cn (T.G.); songrongjing@bjmu.edu.cn (R.S.); xjzhang@bjmu.edu.cn (X.Z.); jinling@bjmu.edu.cn (L.J.); 2 Department of Pharmacology, Ningxia Medical University, Yinchuan 750004, China; E-Mail: lei1153@163.com; 3 Department of Integrated Traditional Chinese and Western Medicine, Peking University, Health Science Center, Beijing 100191, China; E-Mail: leezhixin@tom.com; 4 Department of Laboratory Medicine and Pathobiology, University of Toronto, Toronto, Ontario M5S 1A8, Canada; 5 Beijing N&N Genetech Company, Beijing 100082, China; E-Mail: nngene@sohu.com

**Keywords:** glucokinase (GCK), MODY2, differentially expressed genes, kidney, Gpx3

## Abstract

Liver glucokinase (GCK) deficient mice possess mild renal complications associated with diabetes. To investigate the progression of kidney disease and identify candidate genes involved in the pathogenesis of renal damage, we examined changes in tissue structure and gene expression in the kidneys of liver-specific GCK knockout (gck^w/−^) mice and age-matched normal wild-type control (gck^w/w^) mice as they aged. Suppression subtractive hybridization (SSH) was used to identify candidate genes that showed a pattern of differential expression between kidneys of gck^w/−^ and gck^w/w^ mice at 60 weeks of age. Differential expression of the candidate genes was examined by real-time qPCR in liver-specific gck^w/−^ and gck^w/w^ mice at 16, 26, 40, 60, and 85 weeks of age. Among the candidate genes, only glutathione peroxidase-3 (GPX3) was confirmed to show differential expression by qPCR in the 60-week old mice, however two others genes, MALAT1 and KEG, showed significant changes at other ages. This study shows that liver-specific glucokinase deficient mice display changes in kidney morphology by 40 weeks of age, and that renal complication may be correlated with a reduction in GPX3 levels. Since decreased GPX3 mRNA expression was observed at 26 weeks, which is younger than the age when pathological changes can be seen in kidney biopsies, GPX3 may serve as an early marker for kidney damage.

## 1. Introduction

Diabetes mellitus is a common endocrine disorder characterized by hyperglycemia. It predisposes individuals to chronic complications affecting their eyes, blood vessels, nerves and kidneys. Diabetic nephropathy is one of the major microvascular complications of diabetes and can lead to end-stage renal failure [1]. The late stages of renal disease and the end result for the kidney appear to be similar for both Type 1 and Type 2 diabetes mellitus [2]. While much effort has been put into investigating the development of diabetic nephropathy, its pathogenesis remains unclear [3].

Monogenic diabetes, also called maturity-onset diabetes of the young (MODY), accounts for about 2%–5% of all diabetic patients [4]. Glucokinase diabetes, also known as MODY2, is one of the most prevalent subtypes of MODY and is associated with mutations in the glucokinase (GCK) gene on Chromosome 7p. Heterozygous mutations that inactivate the enzymatic function of GCK from one of the two alleles lead to MODY2, which is characterized by chronic mild hyperglycemia, with an onset usually before the age of 25 [5]. Previously, we constructed and characterized a liver-specific glucokinase gene knockout mouse model of MODY2 using a Cre-loxP gene targeting strategy [6]. Our liver-specific GCK knockout mice were shown to have increased levels of blood glucose from the age of 4 weeks, and had both significantly higher fasting blood glucose levels and impaired glucose tolerance, compared to wild-type gck^w/w^ mice, at 6 weeks [6]. The life span of the liver-specific gck^w/−^ mice is about 2 years, and the long endurance of hyperglycemia may lead to renal damage in these mice.

To investigate the pathogenesis of the renal lesions in the gck^w/−^ mice, we used the suppression subtractive hybridization (SSH) technique to identify genes that are potentially differentially expressed between diabetic and normal kidney tissues, and thus are candidates to be involved in kidney disease.

## 2. Results and Discussion

### 2.1. Changes in Biochemical Parameters in Gck Deficient Mice as They Age

**Figure 1:**
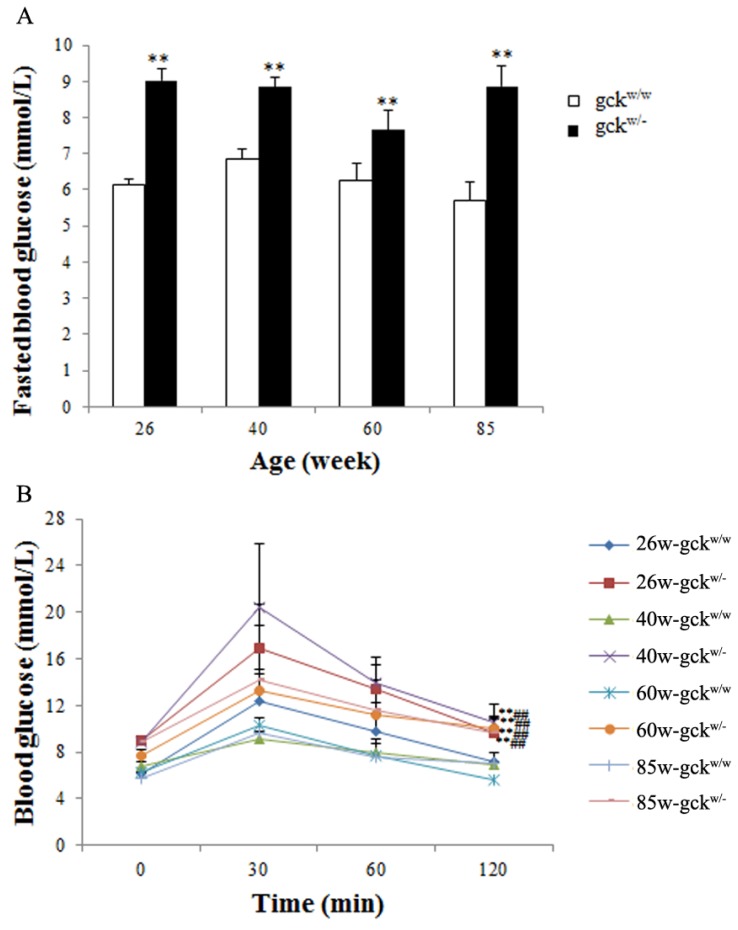
Fasting blood glucose (**A**) and intraperitoneal glucose tolerance tests (IPGTT) curves (**B**) in gck^w/−^ and gck^w/w^ at different ages. Data represent the mean ± SD. (*n* = 6). (**A**) ** *p* < 0.01 *vs.* age-matched gck^w/w^ mice; (**B**) ** *p*< 0.01 *vs.* age-matched gck^w/w^ mice at 0 min; ^##^*p* < 0.01 *vs*. age-matched gck^w/w^ mice at 120 min.

As an initial step in characterizing the effect of aging on gck^w/−^ mice, their fasting blood glucose levels were measured, and it was found that in all age groups, the gck^w/−^ mice had significantly higher levels (*p* < 0.01) (Figure 1A). We next used intraperitoneal glucose tolerance tests (IPGTT) to assess glucose metabolism. Gck^w/−^ mice at ages of 26, 40, 60 and 85 weeks displayed characteristic diabetic curves (Figure 1B), where the blood glucose levels at 120 min were significantly higher than at 0 min (*p* < 0.01), whereas in wild-type mice, the 120-min glucose levels were not different from those at 0 min. Serum triglyceride, total cholesterol and urea nitrogen levels were also examined in the gck^w/−^ and gck^w/w^ mice, but no significant differences were found between the age-matched groups (Table 1). A significant increase in serum creatinine levels, however, was observed in the gck^w/−^ mice at 40 weeks of age (*p* < 0.05), but this transient increase was reduced to wild-type levels at both older ages of 60 and 85 weeks (Table 1). Biochemical changes in urine were also examined. Except for 26-week-old gck^w/−^ mice, the urine protein content of gck^w/−^ mice was significantly higher than that for age-matched gck^w/w^ mice (*p* < 0.01, Table 1). Urine volume showed little change, with only 60-week-old gck^w/−^ mice showing a significantly higher urine volume compared to their age matched controls (*p* < 0.05, Table 1).

### 2.2. Morphological Analysis of Kidney Tissue

**Figure 2:**
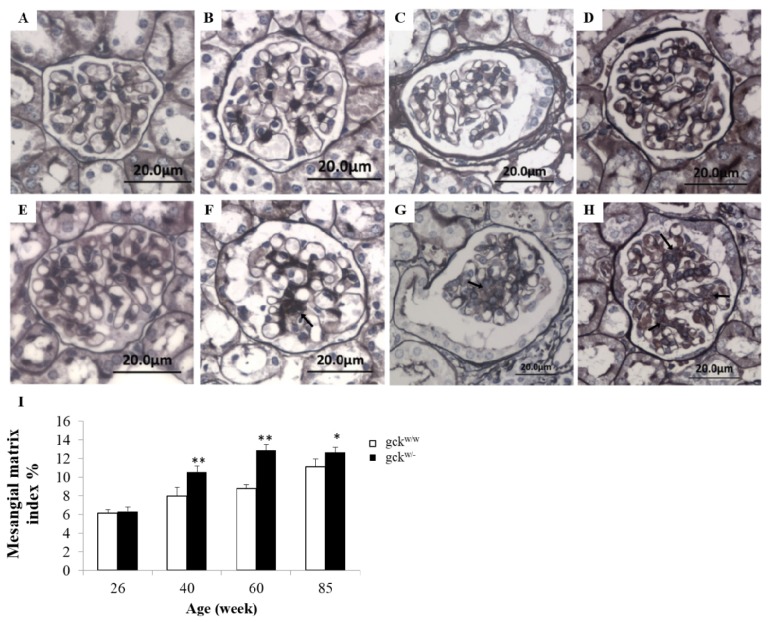
Progression of renal damage in the kidney of gck^w/−^ mice. Glomerular histology of gck^w/w^ mice at 26, 40, 60 and 85 weeks of age (**A**, **B**, **C** and **D**, respectively), and gck^w/−^ mice at 26, 40, 60, 85 weeks of age (**E**, **F**, **G** and **H**, respectively) (six mice (*n* = 6) were examined for each group, with representative slides shown). Tissue was stained with periodic acid-silver methanamin (PASM) with a magnification of ×400. Arrows indicate areas with accumulation of mesangial matrix. **I**: Mesangial matrix index of mice was calculated to quantify differences with age in the gck^w/−^ and gck^w/w^ mice. * *p* < 0.05, ** *p* < 0.01 *vs.* age-matched gck^w/w^ mice.

**Figure 3:**
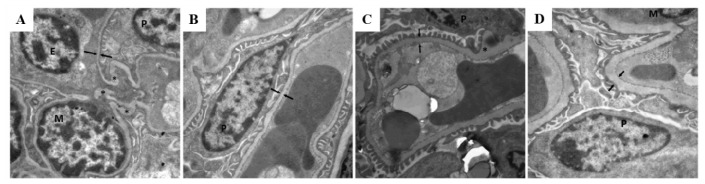

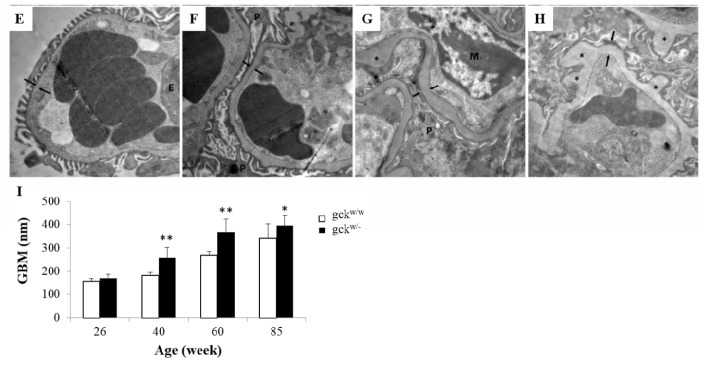
Electron photomicrographs showing age-related growth of glomerular basement membrane (GBM) thickness in gck^w/−^ mice. Electron photomicrographs from gck^w/w^ mice (**A**, **B**, **C** and **D** for 26, 40, 60 and 85 weeks of age, respectively) and gck^w/−^ mice (**E**, **F**, **G** and **H** for 26, 40, 60 and 85 weeks of age, respectively) (six mice (*n* = 6) were examined in each group, with three glomeruli examined per mouse; representative images shown). Magnification is ×12,000. Opposing paired double arrows indicate the GBM. M, E, and P designate mesangial cells, endothelial cells, and podocytes, respectively. * Indicate prominent irregularities in the basement membrane. **I**: Quantification of the GBM thickness. * *p* < 0.05, ** *p* < 0.01 *vs.* age-matched gck^w/w^ mice.

To determine whether gck^w/−^ develop kidney complications, the morphology of kidney tissue was examined. At 26 weeks of age, gck^w/−^ mice show a kidney morphology that is normal in appearance and is similar to that of age-matched wild-type mice (compare Figure 2A,E). As gck^w/−^ age, a gradual increase in the amount of mesangial matrix is observed (see Figure 2F–H for gck^w/−^ mice at 40, 60, and 85 weeks, respectively) compared with their age-matched controls (Figure 2B–D at 40, 60, and 85 weeks, respectively). These results show that renal lesions start at about 40 weeks of age in gck^w/−^ mice, and that these lesions gradually become more aggravated with age. The thickness of the glomerular basement membrane (GBM) shows a similar pattern to the mesangial matrix (Figure 3I). No change in GBM thickness was observed in 26-week-old gck^w/w^ compared with gck^w/−^ mice (Figure 3A,E), however, at 40 weeks of age gck^w/−^ mice showed significantly thicker GBM compared with their age-matched wild-type mice (compare Figures 3B,F, statistical analysis in Figure 3I). The GBM thickness in gck^w/−^ mice continued to grow larger at older ages (Figure 3).

### 2.3. Identification of Differentially Expressed Genes by SSH

Using the SSH technique a total of 450 kidney cDNA clones were obtained (153 and 297 from the reverse and forward subtractions, respectively) from gck^w/−^ mice at 60 weeks of age. After confirming the presence of an insert by double enzyme digestion, 212 of the forward and 117 of the reverse subtraction clones were selected for sequencing, and their sequences were compared to the National Center for Biotechnology Information (NCBI) database using the basic local alignment search tool (BLAST) algorithm. Ultimately, 13 over-expressed (represented by 152 clones) and 12 under-expressed (represented by 71 clones) genes were identified (Tables 2 and 3, respectively).

### 2.4. Expression Profiles of Six Candidate Genes

Our SSH approach identified a number of genes that are potentially over- or under-expressed, however any change in expression needs to be confirmed. Genes represented by the large numbers of clones are the best candidates for genes that show a real change in expression, therefore we chose four of the genes (hepatic nuclear factor 4 alpha (HNF4α), phosphoenolpyruvate carboxykinase 1 (PCK1), metastasis associated lung adenocarcinoma transcript 1 (MALAT1), and kidney expressed gene 1 (KEG1); Table 2) that had the highest number of clones in the over-expression SSH and the two genes (cellular FLICE-like inhibitory protein (c-FLIP) and glutathione peroxidase-3 (GPX3), Table 3) with the most clones from the under-expression SSH experiment. We did not examine ECM1, which was represented by the most clones in the over-expression SSH experiment (Table 2), since its change is likely associated with change in the amount of extracellular matrix [7,8] that was observed in the morphological analysis (Figures 2 and 3) and may be a response to the disease. Among the genes identified by SSH, six candidate genes were chosen for further study: HNF4α (hepatic nuclear factor 4 alpha) is a nuclear transcription factor involved in the regulation of many metabolic pathways such as lipid, amino acid metabolism, and especially glucose metabolism [9–11]. PCK1 (phosphoenolpyruvate carboxykinase 1) encodes the cytosolic isozyme of phosphoenolpyruvate carboxykinase (PEPCK-C), which is the rate-limiting enzyme of gluconeogenesis in the liver and kidney [12,13]. MALAT1 (metastasis associated lung adenocarcinoma transcript 1) is a long non-coding RNA (lncRNA) originally identified as overexpressed in patients at high risk for metastasis of non-small cell lung tumors (NSCLC) [14], however, its role in normal cells remains unknown. KEG1 (kidney expressed gene 1) encodes a glycine *N*-acyltransferase-like protein and is mainly expressed in the kidney [15], where its function is far from clear. GPX3 (glutathione peroxidase-3), which was selected as one of the down-regulated candidate genes, is an extracellular member of the glutathione peroxidase family and has the capacity to protect cells and enzymes from oxidative damage [16]. c-FLIP (cellular FLICE-like inhibitory protein) is a non-redundant antagonist of Caspases-8 and -10 and acts as an inhibitor of apoptosis [17]. HNF4α, PKC1, GPX3, and c-FLIP have roles in metabolic regulation, glucose homeostasis, oxidative stress, and apoptosis, respectively, thus could easily contribute to kidney disease, while MALAT1 and KEG have unknown functions, and may represent novel pathways involved in the disease.

**Figure 4:**
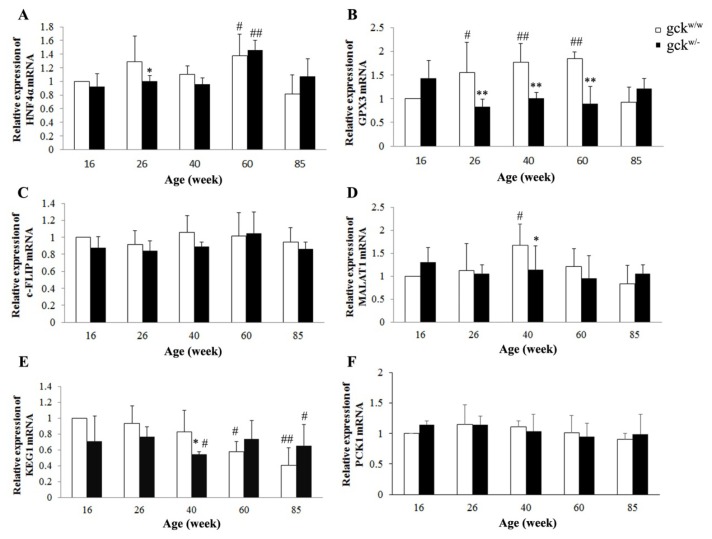
Expression levels of six candidate genes in gck^w/−^ and gck^w/w^ as they age. Expression levels of (**A**) hepatic nuclear factor 4 alpha (HNF4α); (**B**) glutathion peroxidase-3 (GPX3); (**C**) cellular FLICE-like inhibitory protein (c-FLIP); (**D**) metastasis associated lung adenocarcinoma transcript 1 (MALAT1); (**E**) kidney expressed gene 1 (KEG1) and (**F**) phosphoenolpyruvate carboxykinase 1 (PCK1) mRNA levels were examined in the kidney of gck^w/−^ and gck^w/w^ mice at different ages by qPCR. Data represent mean ± SD. (*n* = 6). * *p* < 0.05, ** *p* < 0.01 *vs.* age-matched gck^w/w^ mice. ^#^*p* < 0.05, ^##^*p* < 0.01 *vs.* 16 weeks gck^w/w^ mice.

To confirm changes in expression of our six candidate genes that showed large changes by SSH we performed a qPCR analysis (Figure 4) with β-actin used as an endogenous control. In contrast to expectations based on the SSH analysis, no significant change in mRNA levels was found for five of the six genes (HNF4α, PCK1, MALAT1, KEG1, and c-FLIP) between gck^w/−^ and gck^w/w^ mice at 60 weeks of age (Figure 4). Only GPX3 showed a significant decrease in 60-week-old gck^w/−^ mice compared with age-matched wild-type mice, the result that was expected from the SSH results (Figure 4B).

**Figure 5:**
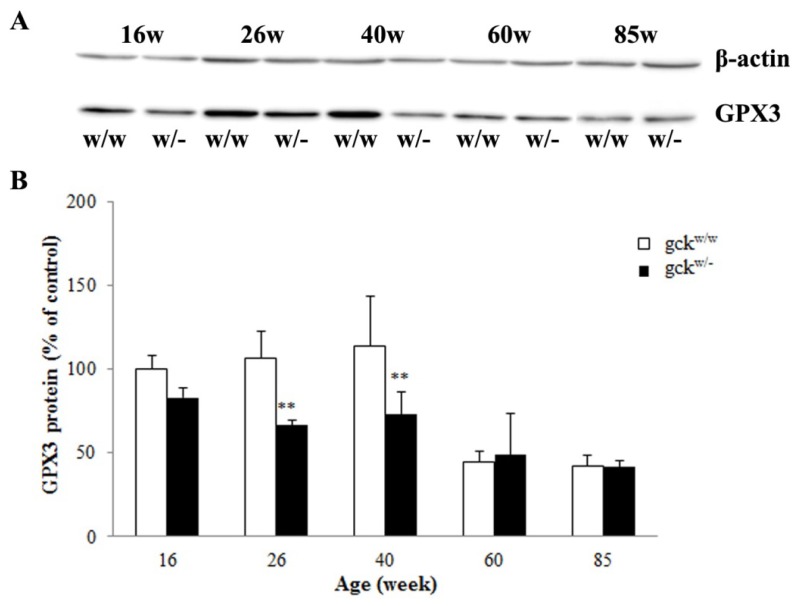
Western blot analysis of GPX3 in the kidney of gck^w/−^ and gck^w/w^ mice at different ages. (**A**) Abundance of GPX3 and β-actin protein extracted from the kidneies of gck^w/−^ (w/−) and gck^w/w^ (w/w) mice at 16 (16W), 26 (26W), 40 (40W), 60 (60W) and 85 (85W) weeks of age was assayed by western blot. β-actin is used as a loading control; (**B**) Quantification of the western blot analysis. Protein levels are compared to that of GPX3 in 16-week-old gck^w/w^ mice (100%) and have been normalized to the β-actin levels. Data represent the mean ± SD. (*n* = 6). ** *p* < 0.01 *vs.* age-matched gck^w/w^ mice.

In addition to the change seen at 60 weeks, GPX3 mRNA levels were also significantly lower in the gck^w/−^ mice at 26 and 40 weeks compared with age-matched wild-type mice (Figure 4B). Changes in the GPX3 protein levels were confirmed by western blot (Figure 5, except 60 weeks).

While the SSH results could not be replicated by qRT-PCR in 60 week old mice, some significant differences in expression were seen at other ages (Figure 4). For HNF4α, expression at 60 weeks was significantly increased for both gck^w/w^ and gck^w/−^ mice compared with 16-week-old gck^w/w^ mice (Figure 4A, *p* = 0.024, *p* = 0.009, respectively). GPX3 mRNA levels of 26, 40, 60-week-old wild-type mice showed an increase compared with 16-week-old gck^w/w^ mice (Figure 4B, *p* = 0.045, *p* = 0.005, *p* = 0.002, respectively). MALAT1 expression in 40-week-old gck^w/−^ mice was significantly lower than for age-matched wild-type mice, and in 40-week-old gck^w/w^ mice it was significantly higher than in 16-week-old wild-type mice (Figure 4D, *p* = 0.031). The expression pattern of KEG1 demonstrated that 40-week-old gck^w/−^ mice had lower expression than age-matched wild-type mice (Figure 4E). However, no significantly change was seen in the expression of c-FLIP or PCK1 between genotypes or with age (Figure 4C,F).

### 2.5. Serum and Renal Gpx Activities

**Figure 6:**
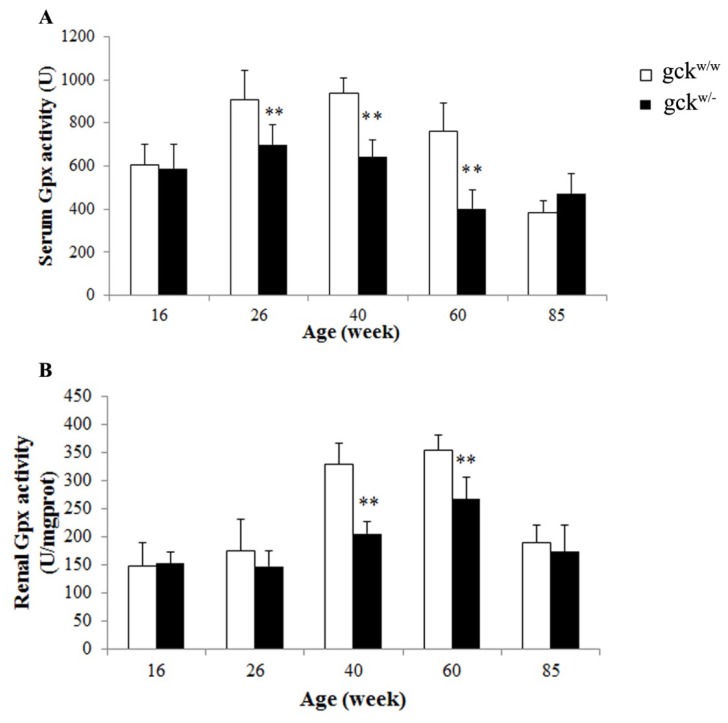
Gpx activities in the serum and kidneys of gck^w/−^ and gck^w/w^ mice at different ages. (**A**) Serum and (**B**) renal Gpx activities were measured in gck^w/−^ mice and gck^w/w^ mice at 16, 26, 40, 60 and 85 weeks of age. Data represent mean ± SD. (*n* = 6). ** *p* < 0.01 *vs.* age-matched gck^w/w^ mice.

Since GPX3 mRNA (Figure 4) and protein levels (Figure 5) changed between gck^w/−^ and gck^w/w^ mice at 60 weeks of age, we tested the levels of Gpx activity. The levels of Gpx activity in serum were significantly lower in gck^w/−^ mice at 26, 40, 60 weeks of age compared with age-matched wild-type mice (Figure 6A). Identical results were obtained for kidney Gpx enzyme activities from kidney extracts except in the 26-week-old mice (Figure 6B).

### 2.6. Antioxidative Capacity of Serum and Kidney in Gck^w/−^ and Gck^w/w^ Mice

**Figure 7:**
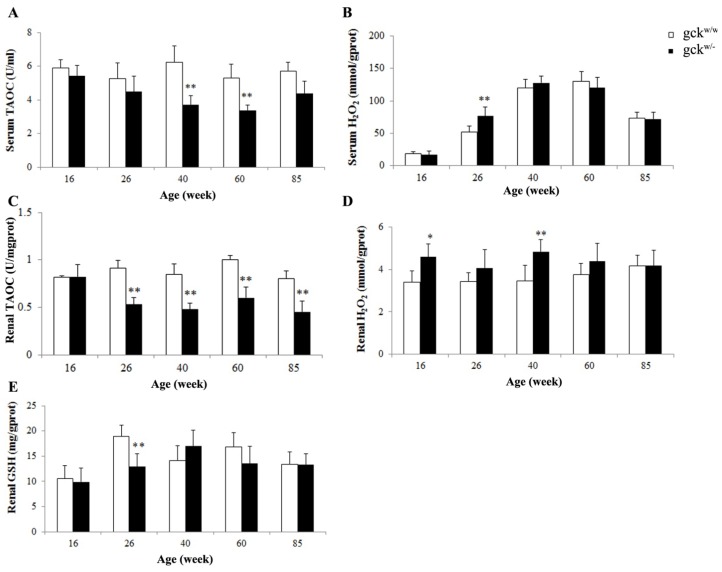
Change in oxidative stress in the serum and kidneys of gck^w/−^ and gck^w/w^ mice as they age. Levels of (**A**) serum total antioxidant capacity (TAOC); (**B**) serum hydrogen peroxide (H_2_O_2_); (**C**) renal TAOC; (**D**) renal H_2_O_2_ and (**E**) renal glutathione (GSH) in gck^w/−^ mice and gck^w/w^ mice at 16, 26, 40, 60 and 85 weeks of age. Data represent mean ± SD. (*n* = 6). * *p* < 0.05, ** *p* < 0.01 *vs.* age-matched gck^w/w^ mice.

Since GPX3 is an antioxidative enzyme, changes in GPX3 levels likely have an impact on the oxidative stress. To examine this, we measured the total antioxidant capacity (TAOC) levels of both serum and the kidneys of gck^w/−^ and gck^w/w^ mice as they aged. TAOC levels in gck^w/−^ mice were significantly lower than in age-matched wild-type mice at 40, 60 and 85 weeks of age (Figures 7A,C). TAOC levels in the kidneys of 26-week-old gck^w/−^ mice were also decreased, but not to statistical significance, compared with age-matched wild-type mice. Levels of hydrogen peroxide (H_2_O_2_), an oxidant, in the serum were higher only at the age of 26-week-old gck^w/−^ when compared with their age-matched wild-type mice (Figure 7B), while in the kidneys, hydrogen peroxide levels in both 16 and 40-week-old gck^w/−^ mice were higher than those for age-matched wild-type mice (Figure 7D). The concentration of glutathione (GSH) in the kidneys was measured and revealed that gck^w/−^ mice had lower GSH levels than age-matched control mice at 26 weeks of age (Figure 7E).

### 2.7. Discussion

Glucokinase diabetes, MODY2, is one of the most common subtypes of maturity-onset diabetes of the young (MODY) and is caused by mutations in the gene encoding glucokinase. Several transgenic rodent models of this disease have been developed, including global and tissue-specific gck-gene knockouts, to help understand the role of GCK in glucose homeostasis at the cellular level [18–20]. Homozygous global GCK knockout results in perinatal death caused by severe diabetes [18,19]. Similarly, homozygous human GCK gene mutations result in a severe diabetes named neonatal diabetes mellitus [21]. Homozygous GCK β-cell specific knockout mice show severe hyperglycemia and death by postnatal day 4, while heterozygous β-cell or liver-specific GCK knockout mice survive with mild diabetes [18–20]. The heterozygous tissue-specific mouse models recapitulate many of the features of the GCK-MODY phenotype seen in humans [4,6,19]. Clinical studies of patients with MODY2 show that while they have persistent mild fasting hyperglycemia, their glucose intolerance remains stable in long-term follow-up studies [22,23]. Animal models of MODY2 show mild hyperglycemia, as seen in MODY2 patients [24], however, few studies have focused on the impacts associated with a long-term exposure to a defect in glucokinase activity. Our study focused on the renal complications that result from long-term mild hyperglycemia caused by a GCK defect and found that mild hyperglycemia can cause renal damage. In our model, a diabetic state developed in the gck^w/−^ mice by the age of 4 weeks, based on the observation of increased fasting blood glucose levels [6], with fasting blood glucose levels that were significantly higher than those of age-matched wild-type mice up until an age of 85 weeks (Figure 1A). The GCK defect also lead to impaired liver glycogen synthesis, which may result in the abnormal IPGTT curves seen in the gck^w/−^ mice (Figure 1B).

Measurements of serum triglyceride and total cholesterol concentrations (Table 1), which showed no difference between knockout and wild-type mice, indicated that the liver-specific GCK deletion leads to a state of mild diabetes and may have no effect on lipid metabolism. Serum urea nitrogen and creatinine levels are two commonly used measures of kidney function. The concentrations of urea nitrogen and creatinine showed no difference between the knockout and wild-type mice except the creatinine levels in 40-week-old gck^w/−^ mice where they showed a transiently higher level of serum creatinine compared with age-matched wild-type mice (Table 1). While the increase of serum creatinine in 40 weeks gck^w/−^ mice was transient, it attracted our attention, as mild hyperglycemia may cause kidney complications. Creatinine is a waste product generated by the degradation of creatine in muscle, therefore its levels should depend upon muscle mass [25]. Muscular young or middle-aged adults may have more creatinine in their blood than the norm for the general population, while older individuals may have less creatinine in their blood [25]. Since serum creatinine is an important indicator of kidney function, elevated creatinine levels may signify impaired kidney function or kidney disease, however, no uniform standard exists for mouse serum creatinine levels. The normal clinical range for creatinine in humans is 44–106 μM, with levels > 530 μM being a sign of kidney disease and in some pathological conditions it can exceed 1000 μM [26]. In the 40-week-old gck^w/−^ mice, the increase in serum creatinine levels was less than one-fold, compared with age-matched wild-type mice, suggesting that only slight kidney damage existed in these mice. The serum creatinine level of gck^w/−^ mice was reduced after 60 weeks, which might have been due to the loss of muscle with age. Kidneys have a very strong compensatory ability, and creatinine levels can continue to increase until more than half of the kidney function has been lost. Although kidney damage progressed with age, the renal complications occurring in the gck^w/−^ mice was small, thus the function of kidneys may still have been within the normal range. While changes in creatinine levels may have been minimal, increased concentrations of protein in the urine of gck^w/−^ mice of 40 weeks of age and older, compared with age-matched gck^w/w^ mice (Table 1), indicated that they had kidney damage. Furthermore, PASM staining and electron micrographs of the kidneys of gck^w/−^ mice 40 weeks of age or older illustrated histological evidence for kidney complications that increased with age (Figures 2 and 3). Further studies, measuring water consumption, eGFR, and urine albumin levels, should provide more information on the extent of damage to the kidneys in the gck^w/−^ mice.

SSH was used to identify potential candidate genes involved in kidney disease initiated by mild hyperglycemia. From a total of 450 cDNA clones generated by the two SSH experiments, 13 genes were identified as potentially up-regulated in the 60-week-old gck^w/−^ mice and 12 down-regulated (Tables 2 and 3). Kidney damage can be induced by multiple mechanisms such as metabolic disorders, impaired glucose homeostasis, oxidative stress, and cellular apoptosis, and the genes HNF4α, PCK1, GPX3 and c-FLIP, respectively, identified by SSH, may have roles in these mechanisms. As MALAT1 and KEG1 are novel genes with unclear functions, we focused on these genes to determine if they contribute to novel pathways in the development of kidney complications. When real-time qRCR was used to confirm changes in expression, c-FLIP (Figure 4C) and HNF4α (Figure 4A) were found not to have a significant difference between the knockout and wild-type mice at 60 weeks of age. Although expression of MALAT1 and KEG1 were similar between 60-week-old knockout and wild type mice, lower levels of expression were seen in the knockout mice compared to age-matched wild-type mice at 40 weeks of age (Figure 4D,E). Serum creatinine was increased at the age of 40 weeks, but returned to normal levels at 60 and 85 weeks (Table 1). Kidney lesions due to diabetic nephropathy are irreversible and, although blood glucose control or other therapies may delay the progression to kidney failure, kidney function cannot be restored. We therefore conclude that at an age of 40 weeks specific changes have occurred in the gck^w/−^ mice that cause kidney damage as detected by morphology analysis, with KEG1 and MALAT1 possibly being responsible.

Glutathione peroxidase 3 is an extracellular member of the glutathione peroxidase family [16] and is primarily secreted by the kidney proximal convoluted tubule cells. It binds specifically to basement membranes of the mouse renal cortex tubule cell [27]. Chronic renal failure sharply reduces plasma glutathione peroxidase activity, while kidney transplantation restores the level of enzyme activity [28]. Glutathione peroxidases (GPXs) are major antioxidative enzymes that catalyze the reduction of H_2_O_2_, organic hydroperoxides and lipid peroxides by using glutathione as a reductant [29–33]. In mammals, eight glutathione peroxidases (Gpx1-Gpx8) have been identified to date, with Gpx3 being the only glutathione peroxidase secreted into the plasma, which has led to it being named plasma glutathione peroxidase [34]. As expected, the level of GPX3 was decreased in the 60-week-old gck^w/−^ mice, with 26 and 40-week-old mice showing a similar result (Figure 4B). The protein levels for Gpx3 do not correlate perfectly with its mRNA levels (Figure 5, groups at 60 weeks), raising the possibility that GPX3 expression is post-transcriptionally regulated. Further research is needed to address this issue. Glutathione peroxidase family members catalyze the same reaction to reduce the H_2_O_2_ and organic hydroperoxide levels: 2GSH + H_2_O_2_ → GSSH + 2H_2_O, 2GSH + ROOH → GSSH + 2H_2_O (ROOH, organic hydroperoxides), therefore, measurement of Gpx activity levels may include the activities of several glutathione peroxidases. Since Gpx3 is the only GPX secreted into plasma, the serum Gpx activity should represent only Gpx3 activity. In the kidney, however, Gpx1 and Gpx4 are also expressed [35], thus three enzymes contribute to Gpx activity levels. This complexity in GPX activity may explain why kidney Gpx activity levels are not consistent with serum Gpx activity at 26 weeks of age (Figure 6B). TAOC levels in gck^w/−^ mice, in both serum and the kidney, show a reduction at 40, 60 and 85 weeks of age, compared with age-matched wild-type mice, in addition to renal TAOC at 26 weeks of age (Figure 7A,C). The reduction of TAOC reveals dysfunction in the antioxidative system in gck^w/−^ mice. Since gluthathione peroxidases are the major antioxidative enzymes, the decreased levels of GPX3 expression may account for the reduction in TAOC levels. Although both TAOC and Gpx activity in gck^w/−^ mice are down-regulated, the mild hyperglycemia state of these mice seems to only induce a slight oxidative stress as only a few of the gck^w/−^ mice age groups show slightly higher H_2_O_2_ levels (Figure 7B, serum H_2_O_2_ at 26 weeks; D, renal H_2_O_2_ at 16 and 40 weeks) and lower GSH concentrations (Figure 7E, renal GSH at 26 weeks) compared with age-matched wild-type mice. Our research suggests that the kidney damage found in gck^w/−^ mice is largely caused by oxidative stress, with decreased GPX3 being the major cause of this process. The levels of GPX3 mRNA and activity both were reduced at an early age (26 weeks), leading to a decline in antioxidant capacity, which we suggest eventually results in mesangial matrix expansion, thickening of the GBM, and proteinuria. Our research indicates that long-term hyperglycemia, due to MODY2, leads to kidney complications, and that the dysfunction of GPX3 may play a key role in this process. Since the decreases in GPX3 mRNA expression, and protein levels, occur earlier than the observed changes in kidney morphology, GPX3 may be an early marker of diabetic kidney disease, and a potential target for therapy to prevent damage.

## 3. Experimental Section

### 3.1. Animals

Liver-specific gck^w/−^ mice were previously created by our lab [6]. Both wild-type and knockout mice are in the C57BL/6J background. Liver-specific heterozygous gck^w/−^ mice at 16, 26, 40, 60 and 85 weeks of age and wild-type control animals (gck^w/w^ mice), were used in our study. In our previous study, gck^w/−^ mice were found to have higher blood glucose levels than their wild-type controls after 4 weeks of age [36]. All animal experiments were conducted in accordance with the “Guidelines for Animal Experiment” and were approved by the Animal Care Committee of the Peking University Health Science Center.

### 3.2. Biochemical Analysis

Body weight was measured in all mice at 26, 40, 60 and 85 weeks of age, with six mice in each group. Blood glucose concentrations were measured, after an 8h fast, from the tails of mice with a Roche blood glucose monitor (Glucotrend 2). An intraperitoneal glucose tolerance test (IPGTT) was performed in each group by intraperitoneal injection of a 20% glucose solution at a dose of 2 g kg^−1^. Animals were tested at 3:00 p.m. after the 8-h fast. Mice were placed in metabolic cages to collect 24-h urine samples for volume measurement. Urine was stored at −70 °C for later analysis. Serum triglyceride, total cholesterol, urea nitrogen and creatinine were determined using commercial kits (Beihua Kangtai, Beijing, China, cat # 6304, cat # 6031, cat # 6302, cat # Y010, respectively) and an automatic analyzer (AMS-18C). Urine protein concentration was determined using a CSF and Urine protein kit (Leadman, Beijing, China, cat # CS8480).

### 3.3. Renal Morphology

After fasting for 8 h, animals were sacrificed by cervical dislocation, and kidneys were quickly removed and weighed, with the left kidney being processed for histological examinations. For examination by light microscopy, kidneys were fixed in 10% formalin for 24 h at room temperature, dehydrated, and embedded in paraffin, and 4-μm-thick sections were stained with periodic Schiff-methenamine (PASM) agent. Digital images of glomeruli were obtained from microscopy at high power (×400). Mesangial matrix index was calculated as the ratio of mesangial area to glomerular tuft area [37]. Six mice were analyzed in each group and 10 glomeruli were selected per mouse. For electron microscopy, kidneys were cut into small tissue blocks (1 mm^3^) and fixed in 2.5% glutaraldehyde fixative with 0.1 mol/L cacodylate buffer, pH 7.4, overnight at 4 °C. Digital images of the GBM thickness were obtained from electron microscope (JEM-1400 Electron Microscope, Tokyo, Japan). GBM thickness was estimated according to the methods described by Sato *et al.*[38], where three glomeruli from each animal were examined, and an average of 15 electron micrographs were taken per glomeruli.

### 3.4. Suppression Subtractive Hybridization (SSH)

SSH were performed with RNA isolated using pooled samples from the kidneys of three 60-week-old gck^w/w^ mice and three 60-week-old gck^w/−^ mice in both directions to identify transcripts that were both up- and down-regulated. To find genes that were overexpressed in 60-week-old gck^w/−^ mice, RNA from these mice was used as the “tester”, while the 60-week-old gck^w/w^ mice RNA was used as the “driver”. For the reverse subtraction analysis, the “tester” and “driver” mice were switched. Kidney total RNA was extracted with TRIZOL (Invitrogen, Carlsbad, CA, USA, cat # 15596-026). PolyA+ mRNA was isolated using the Purification of poly(A) RNA kit (Macherey-Nagel, Düren, Germany, cat # 740655) and converted to double-stranded cDNA using a M-MLV RTase cDNA Synthesis kit (Takara, Dalian, China, cat # D6130). Tester and driver cDNAs were digested with RsaI (Promega, Southampton, UK, cat # R6371). SSH was performed using the PCR-SELECT cDNA Subtraction Kit (Clontech, Palo Alto, CA, USA, cat # 637401) according to the manufactures instructions. PCR reaction products generated by SSH were shotgun ligated into the pMD-19 T vector (Takara, Dalian, China, cat # D102A), transformed into DH5α cells, and plated onto Luria-Bertani (LB) (Sigma, St. Louis, MO, USA) agar plates containing ampicillin (100 μg/mL). Recombinant colonies were picked from the plates and sequenced by Invitrogen. The identity of the clone sequences was determined by BLAST searches against the NCBI mouse refSeq RNA database.

### 3.5. Quantification of mRNA by Real-Time Quantitative PCR

Total kidney RNA was extracted using TRIZOL. PrimerScript^®^ 1st Strand cDNA Synthesis Kits (Takara, cat # D6110A) were used to synthesize cDNA. RNA was isolated from a total of six mice of each genotype at each age. Quantitative detection of the mRNA levels for the β-actin, GPX3, HNF4α, c-Flip, MALAT1, KEG1 and PCK1 genes was performed with an MiniOpticon optical system (Bio-Rad, Hercules, CA, USA) with the iQ™ SYBR Green PCR SuperMix (Bio-Rad, cat # 170-8880) according to the manufacturer’s instructions. Primers for qPCR for each gene (Table 6) were synthesized by Takara. PCR amplification was carried out in a total volume of 20 μL, containing 1 μL cDNA solution, 10 μL of 2 × iQ™ SYBR Green PCR SuperMix, 1 μL each primer at 5 μM, 7 μL of nuclease-free water. β-actin was quantified, and used for the normalization of expression values of the other genes. Fluorescence signals measured during the amplification were considered positive if the fluorescence intensity was more than 20-fold greater than the standard deviation of the baseline fluorescence [39]. The ΔΔ*C*_T_ method of relative quantification was used to determine the fold change in expression [40]. Here, the threshold cycle (*C*_T_) values of the target mRNAs were first normalized to the *C*_T_ values of the internal control, β-actin, in the same samples (Δ*C*_T_ = *C*_Ttarget_ − *C*_Tcon_), and then further normalized with the internal control (16-week-old gck^w/w^ mice were used as internal control) (ΔΔ*C*_T_ = Δ*C*_T_ − Δ*C*_Tcon_). Fold change in expression was then obtained (2^−ΔΔ^*^C^*^T^) as described by Cao *et al*. [41]

### 3.6. Total Protein Extraction and Western Blot

About 50 mg of kidney tissue was homogenized in 300 mL of lysis buffer containing 50 mM Tris-HCl (pH 8.0), 150 mM NaCl, 0.02% NaN_3_, 0.1% SDS, 1 mM EDTA, 100 mg/L PMSF, 1 mg/L leupeptin and 1% NP-40. The mixture was incubated on ice for 30 min, and then the supernatant was removed after centrifuging at 10,000× *g* for 30 min. Protein concentration was determined using the Bradford method. About 50 μg of protein was subjected to sodium dodecyl sulfate polyacrylamide gel electrophoresis (SDS-PAGE) on 12% polyacrylamide gels. Proteins were then electrophoretically transferred to polyvinylidene fluoride (PVDF) membranes (Millipore, Bedford, MA, USA). After transferring, the membranes were blocked 2 h at room temperature with 5% nonfat dry milk in TBS-Tween 20 (TBS-T) followed by incubation overnight at 4 °C with the following primary antibodies: anti-GPx3 antibody (Abcam, Cambridge, MA, USA, cat # ab77410) and anti-β-actin antibody (CST, Danvers, MA, USA, cat # 4967). After three washes in TBS-T, membranes were probed with the appropriate HRP-linked secondary antibodies. The blots were visualized by luminal chemiluminescence ChemiDoc XRS (Bio-Rad) and scanned by Quantity One v 4.6.2 software (Bio-Rad). The protein band density was measured using Quantity One v 4.6.2 software (Bio-Rad). The amount of protein under control conditions was assigned a relative value of 100%.

### 3.7. Measurement of Serum and Renal Gpx Enzyme Activities

Activities of Gpx from serum and kidney homogenates were measured using a commercial kit (Nanjing Jiancheng, Nanjing, China, cat # A005). Protein concentration was determined using BCA protein assay kit (Nanjing Jiancheng, Nanjing, China, cat # 1045-4).

### 3.8. Measurement of TAOC, Hydrogen Peroxide and Glutathione

TAOC and H_2_O_2_ of serum were measured using commercial kits (Nanjing Jiancheng, Nanjing, China, cat # A015 and A064, respectively). TAOC, H_2_O_2_ and GSH of kidney homogenates were determined using commercial kits (Nanjing Jiancheng, China, Nanjing, cat # A015, A064 and A006-1, respectively).

### 3.9. Statistical Analysis

Results are shown as means ± SD. Differences between the control and experimental groups were evaluated by one-way (ANOVA; SPSS 13.0 for Windows, SPSS Inc., Chicago, IL, USA). *p* values less than 0.05 were considered to be statistically significant.

## 4. Conclusions

This study showed that liver-specific glucokinase deficient mice developed kidney damage by 40 weeks of age and that renal complication may be correlated with a reduction in GPX3 levels. A decrease in GPX3 mRNA expression was observed at 26 weeks of age, an age younger than at which changes can be seen in kidney biopsy, which suggests that GPX3 is a potential early marker of kidney damage.

## Figures and Tables

**Table 1:** Biochemical analysis of gck^w/−^ and gck^w/w^ mice at 26, 40, 60 and 85 weeks of age. Data represent mean ± SD. (*n* = 6).

		26 weeks	40 weeks	60 weeks	85 weeks
Body weight (g)	gck^w/w^	36.76 ± 2.64	47.65 ± 1.83	45.11 ± 4.13	39.36 ± 5.37
	gck^w/−^	39.61 ± 4.47	47.55 ± 1.93	47.42 ± 2.63	45.67 ± 4.16
Kidney/body weight (%)	gck^w/w^	1.05 ± 0.12	1.29 ± 0.35	1.07 ± 0.19	1.18 ± 0.19
	gck^w/−^	1.08 ± 0.33	1.22 ± 0.32	1.00 ± 0.08	1.22 ± 0.19
Serum					
Triglyceride (mg/dL)	gck^w/w^	137.75 ± 6.45	107.33 ± 16.46	120.16 ± 37.62	87.67 ± 15.89
	gck^w/−^	123 ± 14.31	106.41 ± 21.64	131.62 ± 26.66	91.00 ± 27.06
Total cholesterol (mg/dL)	gck^w/w^	128.67 ± 20.26	131.11 ± 16.66	147.72 ± 21.44	147.33 ± 17.39
	gck^w/−^	124.40 ± 14.45	137.27 ± 25.05	125.69 ± 13.22	124.25 ± 34.24
Urea nitrogen (mmol/L)	gck^w/w^	7.37 ± 0.19	7.39 ± 0.09	7.16 ± 0.24	6.90 ± 0.07
	gck^w/−^	7.29 ± 0.08	7.37 ± 0.11	7.16 ± 0.11	6.82 ± 0.15
Creatinine (mg/dL)	gck^w/w^	0.73 ± 0.07	0.65 ± 0.11	0.81 ± 0.04	0.74 ± 0.20
	gck^w/−^	0.84 ± 0.10	1.04 ± 0.20 *	0.89 ± 0.19	0.57 ± 0.15
Urine					
Volume (mL/24h)	gck^w/w^	0.65 ± 0.13	2.90 ± 0.37	1.28 ± 0.51	1.73 ± 0.31
	gck^w/−^	0.77 ± 0.21	3.17 ± 0.67	2.07 ± 0.49 *	2.07 ± 0.21
Protein (μg/mL)	gck^w/w^	49.32 ± 9.67	27.52 ± 4.57	20.68 ± 7.41	18.17 ± 2.72
	gck^w/−^	32.42 ± 14.22	152.18 ± 39.54 **	163.63 ± 50.28 **	100.00 ± 18.58 **

* *p* < 0.05,

** *p* < 0.01 *vs.* age-matched gck^w/w^ mice.

**Table 2:** Up-regulated genes isolated by subtractive suppressive hybridization (SSH) from the kidneys of 60-week-old gck^w/−^.

GenBank Accession	Blast result	Number of clones	Function
NM_007899.2	Ecm1, extracellular matrix protein 1	30	Signal transduction
NM_008261.2	Hnf4α, hepatic nuclear factor 4, alpha	27	Transcription regulation
NM_011044.2	Pck1, phosphoenolpyruvate carboxykinase 1, cytosolic	26	Glucose metabolism
NR_002847.2	Malat1, metastasis associated lung adenocarcinoma transcript 1	17	Unknown, long non-coding RNA
NM_029550.4	Keg1, kidney expressed gene 1	13	Transferase
NM_016805.2	Hnrnpu, heterogeneous nuclear ribonucleoprotein U	12	Ribosomal protein
NM_025337.3	Akr7a5, aldo-keto reductase family 7, member A5	9	Oxidoreductase
NM_001081158.2	1300001I01Rik, RIKEN cDNA 1300001I01 gene	7	Unknown
NM_007390.3	Chrna7, cholinergic receptor, nicotinic, alpha polypeptide 7	5	Transporter
NM_001130526.1	Lzts2, leucine zipper, putative tumor suppressor 2	2	Cell cycle
NM_018860.4	Rpl41, ribosomal protein L41	2	Ribosomal protein
NM_021607.3	Ncstn, nicastrin	1	Signal transduction
NM_133218.2	Zfp704, zinc finger protein 704	1	Metal ion binding

**Table 3:** Down-regulated genes isolated by subtractive suppressive hybridization from the kidneys of 60-week-old gck^w/−^.

GenBank accession	Blast result	Number of clones	Function
NM_008161.3	Gpx3, glutathione peroxidase 3	17	Oxidoreductase
NM_145758.1	0610010K14Rik, RIKEN cDNA 0610010K14 gene	15	Unknown
NM_207653.3	c-Flip, cellular FLICE-like inhibitory protein	12	Inhibitor of apoptosis
NM_001159571.1	Ephb4, Eph receptor B4	9	Developmental protein
NM_025974.2	Rpl14, ribosomal protein L14	5	Ribosomal protein
NM_025701.4	Trappc5, trafficking protein particle complex 5	3	Transporter
NM_019883.3	Uba52, ubiquitin A-52 residue ribosomal protein fusion product 1	3	Ribosomal protein
NM_010106.2	Eef1a1, eukaryotic translation elongation factor 1 alpha 1	2	Protein biosynthesis
NM_033080.2	Nudt19, nudix (nucleoside diphosphate linked moiety X)-type motif 19	2	Hydrolase
NM_148932.2	Pom121, nuclear pore membrane protein 121	1	Transporter
NM_001168623.1	Znrf1, zinc and ring finger 1	1	Metal ion binding
NM_001159483.1	Rpl19, ribosomal protein L19	1	Ribosomal protein

**Table 3:** Sequences of qPCR primers.

Gene	Sense primer	Antisense primer
HNF4α	GGTCCATGGTGTTTAAGGACGTG	GTCATCAATCTGCAGCTCTTGGAA
GPX3	AACGTAGCCAGCTACTGAGGTCTGA	CTGTTTGCCAAATTGGTTGGAAG
c-FLIP	TGCACAGCAGACGTATCTCACTTG	TGTTCCACGCATACACTTTGTCC
MALAT1	GAAGACAGGAGCGGCAGACA	GCTTCACCACCACATCCGTATG
KEG1	ACTAACTTGGGCAAGGTCAAGCA	GCAGCATGTGTAACATCCAGTGAG
PCK1	GAACTGAGACTCGCCCTATGTG	GTTGCAGGCCCAGTTGTTGA
β-actin	CATCCGTAAAGACCTCTATGCCAAC	ATGGAGCCACCGATCCACA
PCR conditions 50 °C × 5 s, 95 °C × 10 min, 40 cycles of 92 °C × 15 s and 60 °C × 60 s.		
